# Neutrophilic Urticarial Dermatosis With Angioedema: A Diagnostic and Therapeutic Challenge

**DOI:** 10.7759/cureus.91490

**Published:** 2025-09-02

**Authors:** Shaun M Crist, Arham Bin Kashif, Muhammad A Aziz, Andre Obua

**Affiliations:** 1 Internal Medicine, Florida International University, Herbert Wertheim College of Medicine, Miami, USA; 2 Internal Medicine, Dow University of Health Sciences, Karachi, PAK; 3 Pathology, Jackson Memorial Hospital, Miami, USA

**Keywords:** chronic pruritus, colchicine therapy, facial angioedema, histopathology (hp), inflammatory skin disorders, neutrophilic urticarial dermatosis, urticarial lesions

## Abstract

Neutrophilic urticarial dermatosis (NUD) is a rare subtype of neutrophilic dermatoses (NDs), presenting with recurrent erythematous papules and plaques often resistant to conventional therapies and associated with systemic symptoms such as fever and polyarthritis. Here, we report the case of a 32-year-old female with chronic pruritus, hives, and angioedema, unresponsive to antihistamines and progressing to diffuse urticarial lesions. Initial differential diagnoses included chronic urticaria, systemic lupus erythematosus, and Schnitzler syndrome. Laboratory testing showed elevated C-reactive protein and weakly positive antinuclear antibodies (ANA), while histopathology confirmed NUD with neutrophilic infiltrates and absence of vasculitis. Management involved methylprednisolone and cetirizine, with the eventual introduction of colchicine, leading to temporary symptomatic resolution. However, with eventual tapering of methylprednisolone, symptoms began to recur, prompting the use of immunosuppressive therapy. This case underscores the importance of histopathological analysis in diagnosing NUD along with refractory disease management. Further research on the pathogenesis of NUD, including genetic testing and screening for underlying autoinflammatory syndromes, may refine treatment protocols and enhance patient outcomes.

## Introduction

Neutrophilic dermatoses (NDs) are a diverse group of autoinflammatory skin disorders characterized by sterile infiltration of neutrophils into the dermis, epidermis, or subcutaneous tissue, presenting unique diagnostic and therapeutic challenges. These dermatoses include conditions like Sweet's syndrome and pyoderma gangrenosum, with neutrophilic urticarial dermatosis (NUD) emerging as a rare subtype within this category.

First described by Kieffer et al. in 2009, NUD typically presents with recurrent, mildly pruritic, erythematous papules and plaques that resolve within 24-48 hours and are often unresponsive to standard antihistamines and corticosteroids [1, 2]. The pathophysiology of NUD remains partially understood, though research implicates dysregulated cytokine signaling, particularly through interleukin-1 (IL-1), as a significant factor in neutrophil recruitment. This response is often observed in patients with genetic mutations associated with cryopyrin-associated periodic syndrome (CAPS), a rare hereditary autoinflammatory disorder characterized by genetic mutations that lead to excessive IL-1 production and recurrent episodes of fever, rash, and systemic inflammation [3].

Clinically, NUD presents as a chronic or recurrent eruption described as pink or reddish macules, papules, or plaques. These lesions predominantly appear on the trunk, with rare involvement of the face and limbs. Although NUD is commonly mistaken for chronic urticaria, it is distinguished by the lack of response to antihistamines and the frequent association with systemic symptoms. Unlike conventional urticaria, patients may also experience associated arthralgias, fatigue, and flu-like symptoms, further complicating the clinical picture [4]. Notably, very few cases of NUD have been linked to angioedema, further distinguishing it from conventional urticaria. Dermoscopic findings often show a dense perivascular neutrophilic infiltrate within the dermis with diffuse erythema without fibrinoid necrosis of vascular walls [4].

General management of NUD is often challenging due to its resistance to conventional urticaria treatments. Treatment strategy can vary depending on the presence of underlying systemic disease, including Schnitzler syndrome (SS), CAPS, systemic lupus erythematosus (SLE), or adult-onset Still disease (AOSD), further complicating treatment selection. This is because each associated systemic disease has distinct pathogenic mechanisms, clinical manifestations, and therapeutic responses [4]. For example, NUD in the context of SLE often fails to respond to immunosuppressants typically used for lupus flares, instead requiring neutrophil migration inhibitors such as colchicine or dapsone. In SS or CAPS, IL-1 antagonists like anakinra are the most effective, producing rapid symptom control but requiring continuous administration to maintain response [4]. As a result, treatment must be individualized to address both the cutaneous symptoms and the systemic inflammatory process driving the disease. 

First-line therapy for treatment of NUD typically includes systemic corticosteroids, which may provide symptom relief but often fail to achieve long-term disease control and have a high side effect profile. Additional options include neutrophil inhibitors such as colchicine or dapsone. In refractory cases, immunomodulating drugs, such as IL-1 inhibitors and tumor necrosis factor-alpha inhibitors, may be required for adequate disease management [3].

This report highlights a rare presentation of NUD with angioedema, complicating the diagnostic approach. Additional concerns arise when the disease is refractory to the usual treatment, necessitating the use of medications with a larger side effect profile. This makes it difficult for the patient to achieve long-term symptomatic remission without exposing the patient to additional risks. This case highlights the need for accurate histopathological diagnosis and targeted therapeutic approaches to improve patient outcomes.

## Case presentation

**Figure 1:**
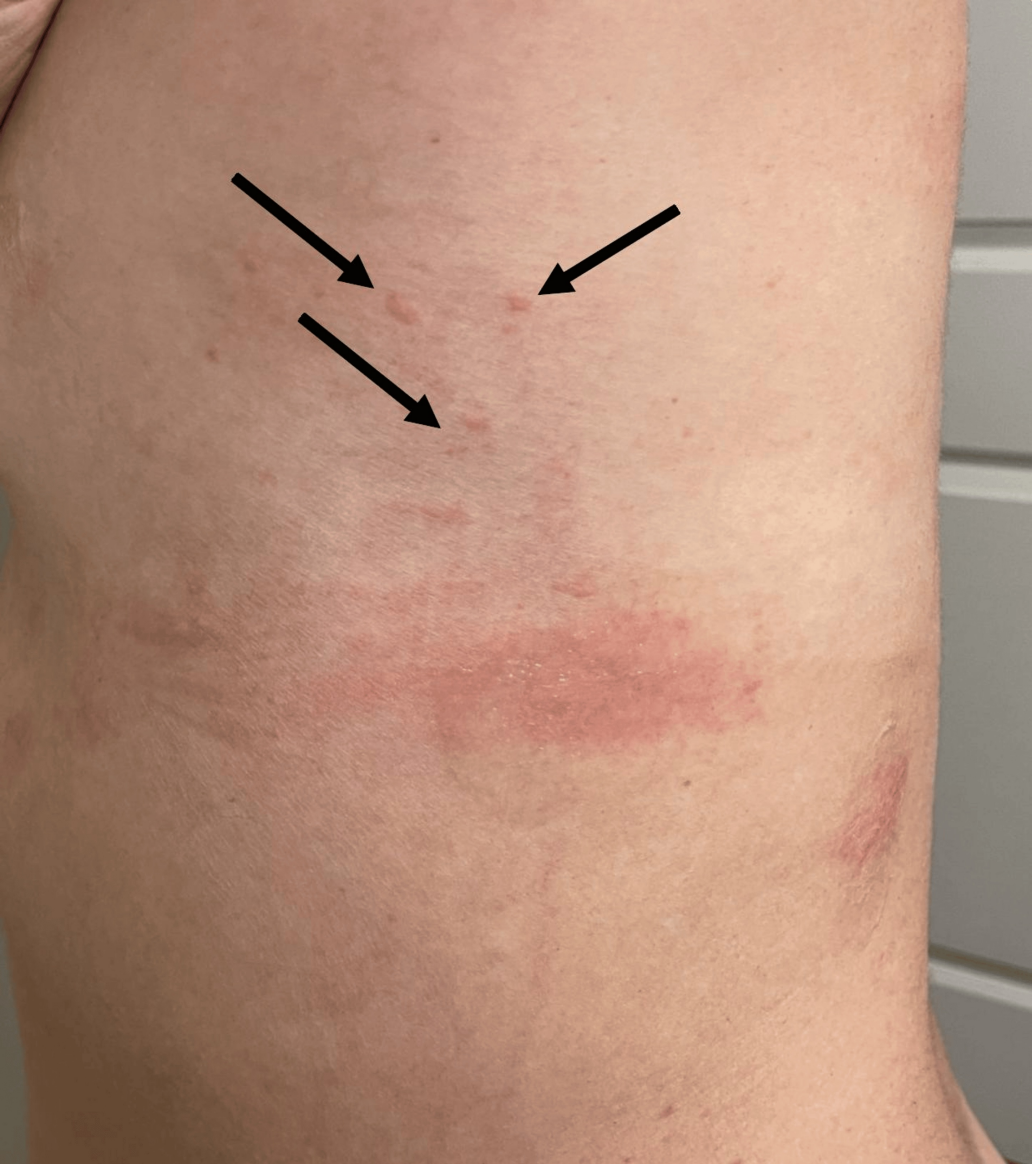
Rash located on the left thorax along the mid-axillary line. The rash appears as multiple erythematous macules and papules with a mildly irregular distribution. The lesions are relatively small, slightly raised, and red in color, indicating inflammation or irritation of the skin.

The patient, a 32-year-old female with no significant medical history, initially presented to the emergency department with complaints of chronic itching, hives, and angioedema. Symptoms began eight months prior to presentation without any identifiable inciting event, starting with intermittent pruritus localized to the chin and left shoulder. She was initially prescribed Montelukast 4 mg for one week, which resulted in the complete resolution of symptoms for one month. Over the next seven months, the patient reported five additional episodes of pruritus with progressive symptoms. A rash described as small, erythematous papules developed and would resolve within 1-2 weeks, becoming more diffuse as each new episode would occur. The rash was reported to have spread from the chin to the left shoulder, and eventually to the trunk (Figure 1), thighs, and upper extremities. The patient noticed that the rash was becoming progressively inflamed, with areas of hyperpigmentation after each episode. During this time, she also reported developing a burning sensation with associated warmth over the affected areas. Cold showers and ice packs would provide relief, while scratching the rash would exacerbate the symptoms. The patient reported no temporal associations to the development of the rash but confirmed photosensitivity. Over this time, treatment with additional Montelukast offered no relief.

**Figure 2:**
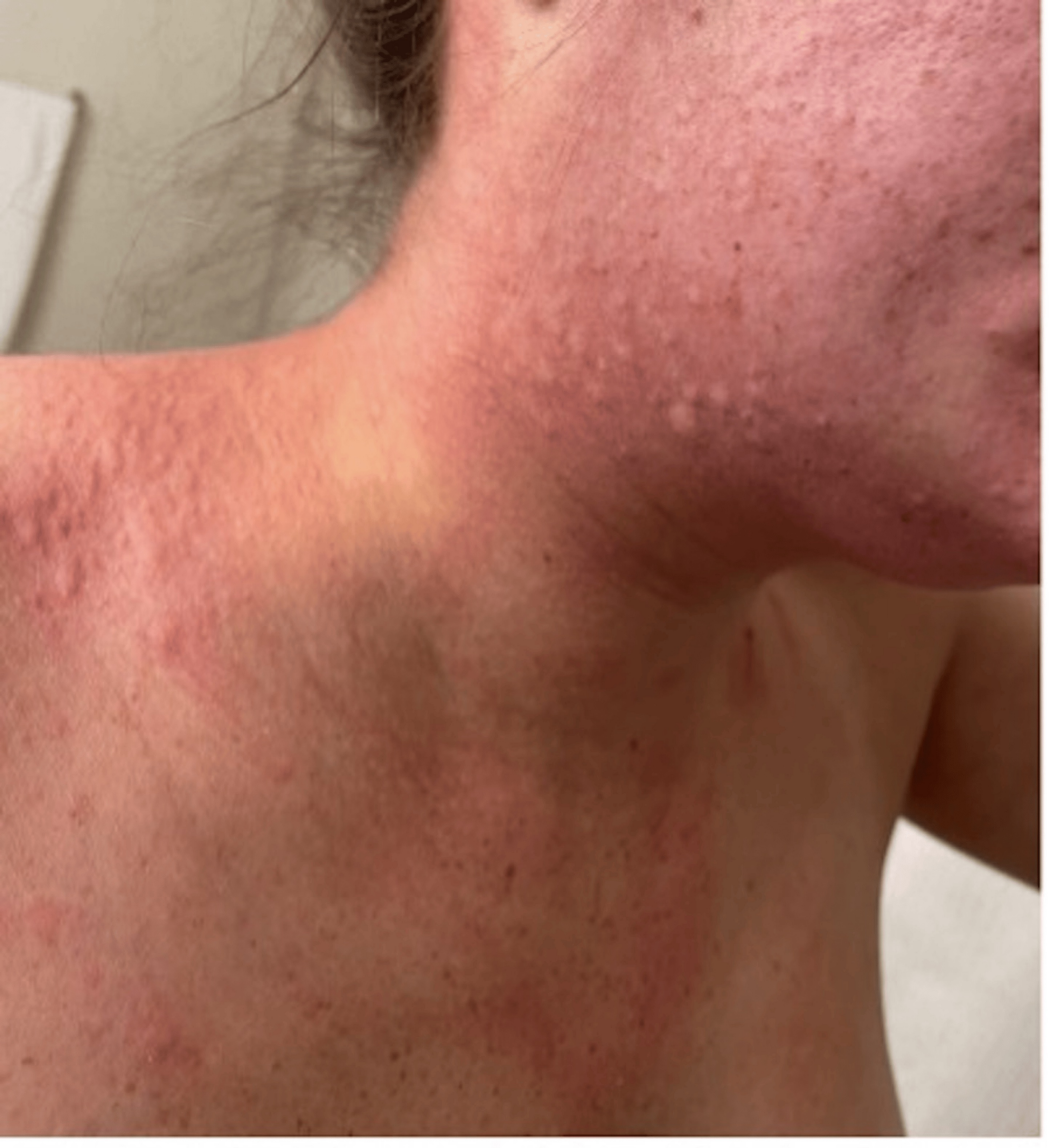
Rash in the buccal area, extending to the neck and upper chest. The skin is diffusely erythematous with visible inflammation and numerous small papules. The redness is most intense along the cheek and neck. The surrounding skin also shows mild redness, extending beyond the primary rash area.

Eight months following the initial eruption, the patient reported more severe symptoms, with the initial rash progressing to diffuse wheals, affecting the upper extremities, cervical region, buccal area (Figure 2), and trunk. She was subsequently prescribed 2.5% topical hydrocortisone cream, which provided no relief after two weeks of treatment in the outpatient setting.

**Figure 3:**
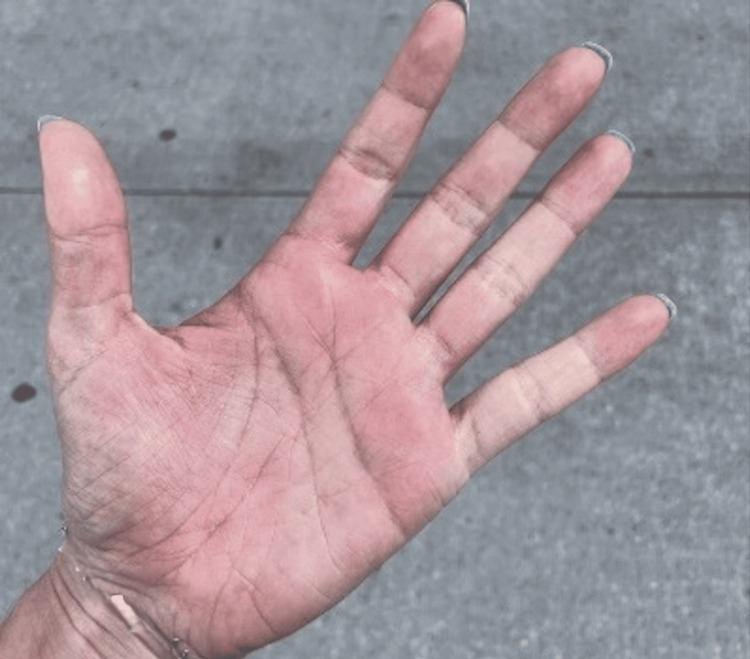
Palmar aspect of the left hand with a notable diffuse erythematous discoloration. The erythematous area lacks sharply demarcated borders, presenting instead as a gradually blended region of discoloration. There is no evident swelling, hyperpigmentation, or hypopigmentation associated with the erythema.

Without any symptomatic improvement, the patient returned to the emergency department with further progression of the rash to involve both hands bilaterally (Figure 3). Additionally, she exhibited new-onset swelling of the upper lip and reported joint pain localized to the wrists and the metacarpophalangeal (MCP) and proximal interphalangeal (PIP) joints of all digits bilaterally.

**Figure 4:**
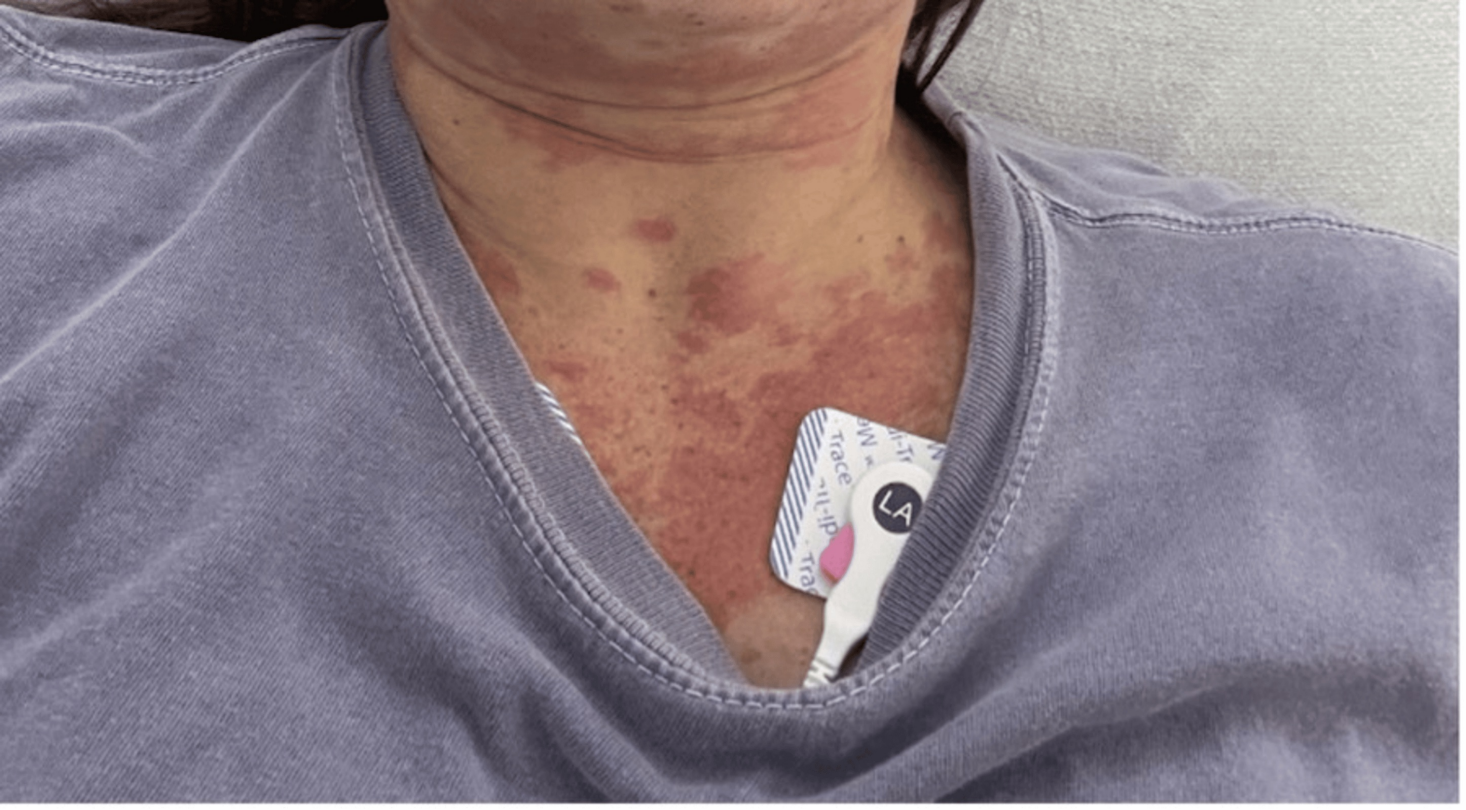
Anterior chest and lower neck area, displaying a widespread erythematous rash covering the central chest and extending upwards onto the lower neck. The rash consists of irregularly shaped, mildly confluent red patches and macules, some of which have a faint, mottled appearance. There are no visible vesicles, pustules, or crusting within the affected area.

**Figure 5:**
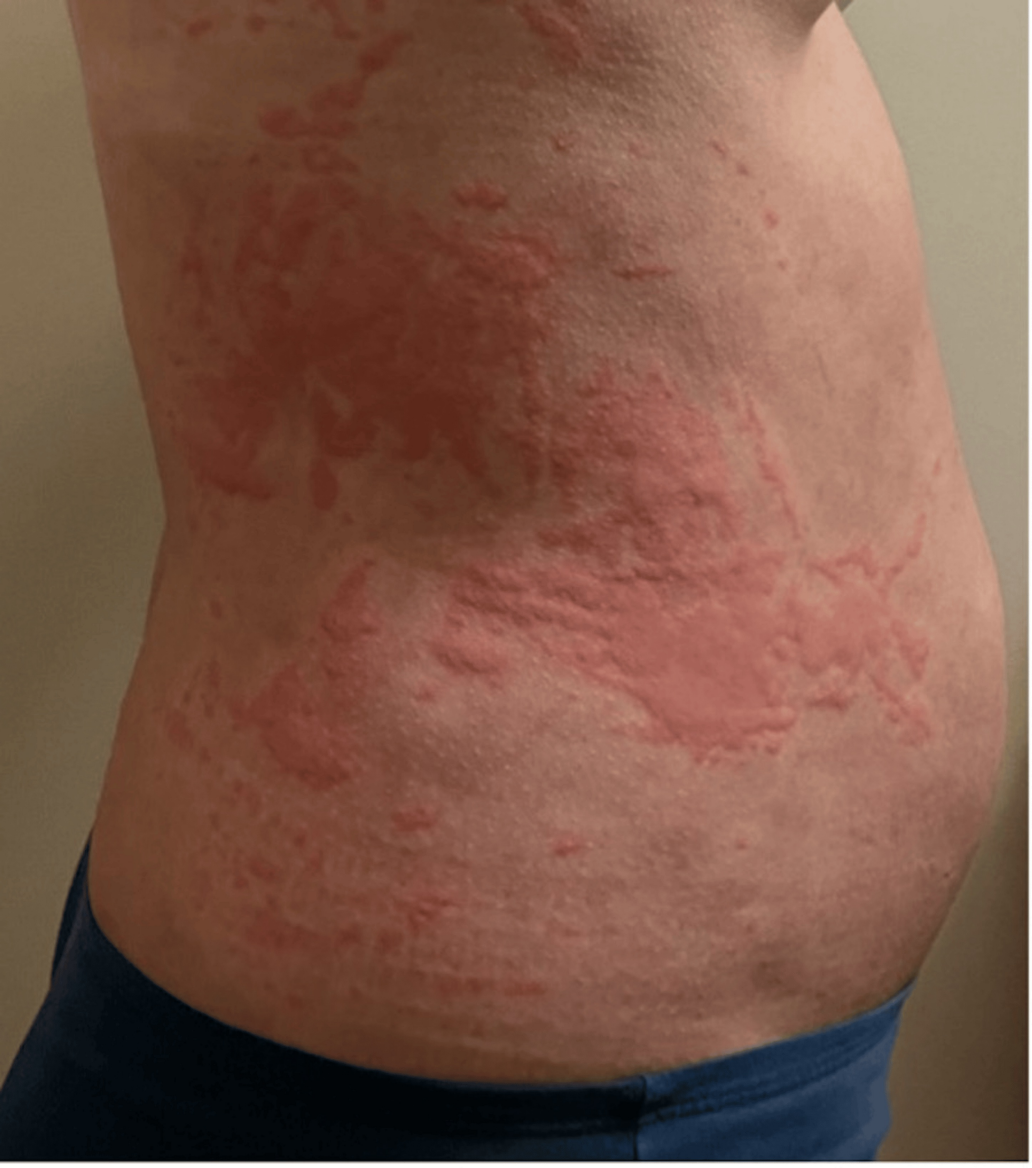
Patient’s torso with erythematous, raised patches on the skin. The lesions are irregularly shaped, slightly elevated, and appear to cover a significant portion of the side of the abdomen and flank. The affected areas have a blotchy appearance with variable redness, typical of urticarial eruptions without visible blistering, scaling, or ulceration.

**Figure 6:**
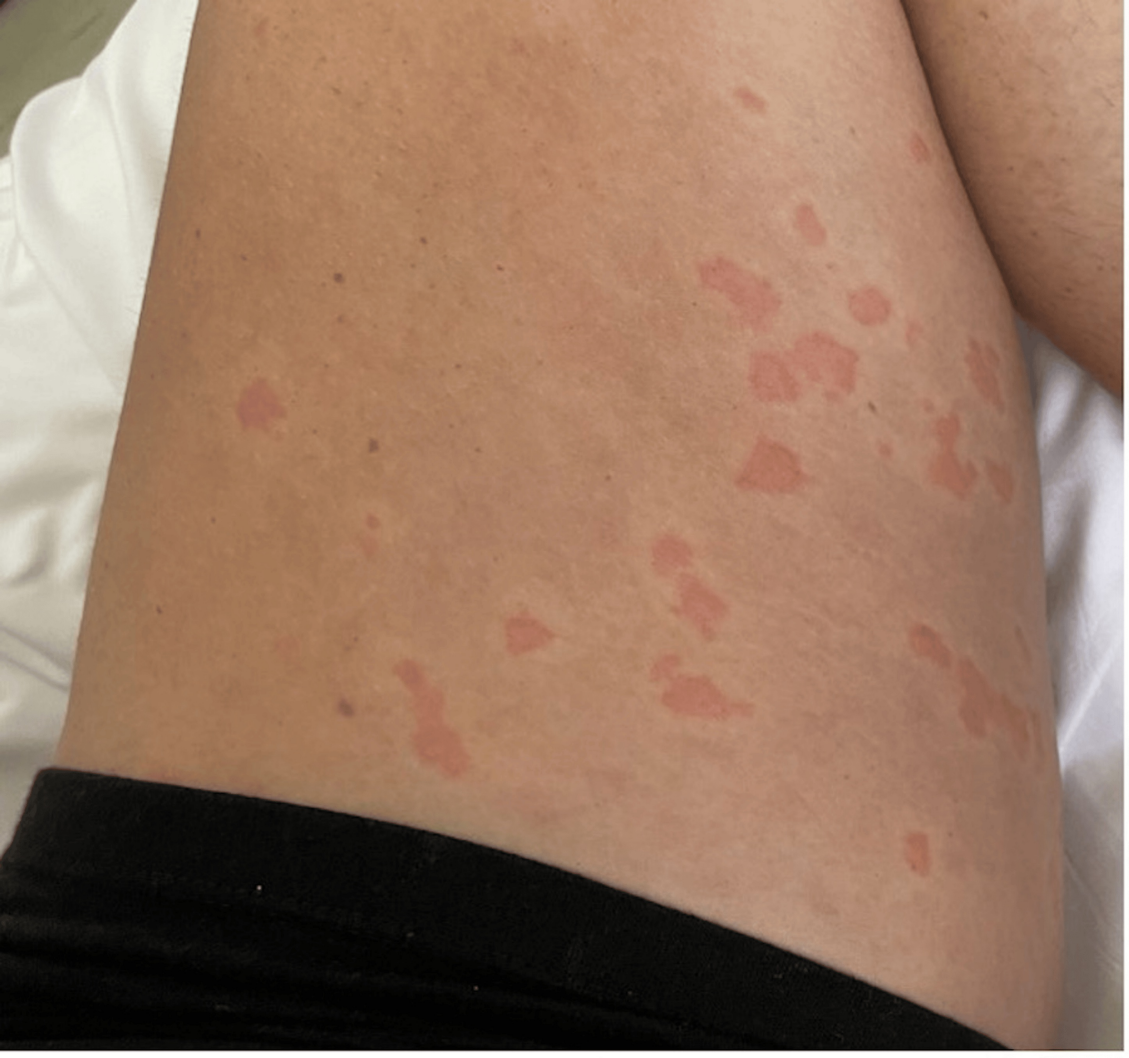
Patient’s upper left thigh with scattered erythematous macules and patches. The lesions are small, irregularly shaped, and relatively flat, indicating mild inflammation without significant elevation or swelling. The affected areas are reddish and slightly lighter in color compared to more intense urticarial reactions, and they appear non-scaly, without blistering or ulceration. This presentation is consistent with a milder manifestation of neutrophilic dermatosis.

Upon presentation, the patient was afebrile, normotensive, and in moderate discomfort. Initial examination revealed non-pitting edema of the upper and lower lips, while conjunctivae and oropharynx were clear, and there was mild tongue edema. The cervical region revealed a diffuse urticarial rash with erythema without notable lymphadenopathy (Figure 4). Mildly edematous, diffuse erythematous, blanching papules and plaques were observed, most pronounced on the abdomen, thighs, upper back, and chest (Figures 4-6). 

**Figure 7:**
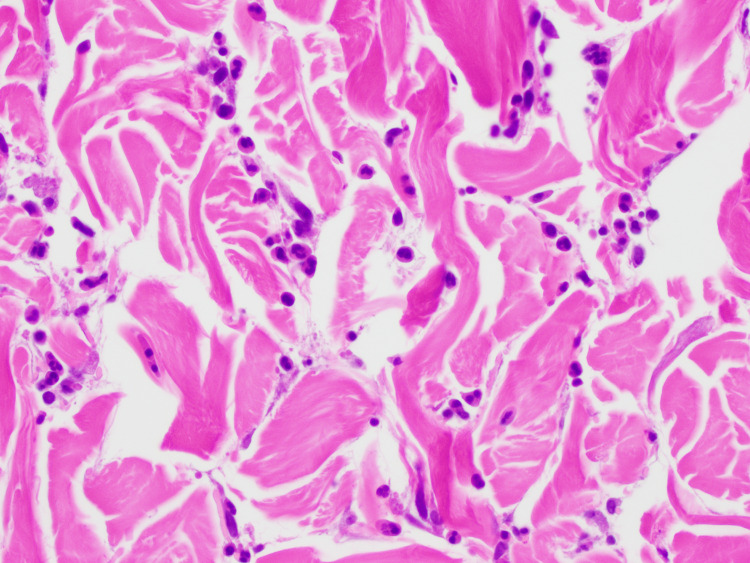
40x microscopic view of a skin biopsy with findings consistent with neutrophilic urticarial dermatosis. The tissue is stained with hematoxylin and eosin, revealing dense infiltrates of neutrophils dispersed throughout the dermis. There is no significant evidence of vasculitis. The surrounding collagen appears disrupted, suggesting inflammation without damage to the blood vessels.

Laboratory testing was significant for an elevated C-reactive protein (CRP) of 4.3 and a weakly positive anti-nuclear antibody (ANA), while other autoimmune, infectious, and immunologic panels, Coombs’ testing, and flow cytometry were unremarkable. Testing for hepatitis and HIV was negative. Diagnostic procedures included a CT chest without contrast, abdominal ultrasound, and a skin punch biopsy of a wheal located on the left scapula with direct immunofluorescence. The biopsy revealed findings suggestive of NUD (Figure 7).

Differential diagnoses for the patient’s presentation included chronic spontaneous urticaria with angioedema, urticarial vasculitis, systemic lupus erythematosus (SLE), Still’s disease, and Schnitzler syndrome due to overlapping clinical features with these conditions. Chronic spontaneous urticaria with angioedema was considered because of the patient’s recurrent hives and swelling episodes, which are hallmark features of this condition.

Urticarial vasculitis was included in the differential due to the persistence and systemic symptoms often associated with vasculitic processes, such as fever and arthralgia. Systemic lupus erythematosus was a consideration given the potential for systemic autoimmune diseases to present with urticarial lesions alongside systemic manifestations such as fatigue, fever, or joint involvement. Still’s disease, characterized by fever, rash, and systemic inflammation, was considered, given the patient’s systemic inflammatory symptoms. Finally, Schnitzler syndrome, a rare autoinflammatory disorder, was included because of its association with chronic urticarial lesions, intermittent fever, and systemic inflammation, often with monoclonal gammopathy. However, the patient’s symptoms did not fit the diagnostic criteria for any of these conditions.

Ultimately, the diagnosis of NUD was made based on histopathological findings of dense neutrophilic infiltration in the dermis without evidence of vasculitis, which is the defining characteristic of this rare condition. This diagnosis explained the patient’s clinical presentation and distinguished it from the other conditions considered in the differential diagnosis.

Following hospital admission, the patient was started on IV methylprednisolone at a dose of 20 mg once daily and diphenhydramine 25 mg IV every eight hours. Two days post-admission with minimal improvement, the corticosteroid regimen was adjusted to a scheduled 20 mg IV dose of methylprednisolone administered three times daily. Additionally, cetirizine was introduced at a dose of 10 mg twice daily. Following a temporary hold on methylprednisolone to trend leukocyte levels, a transient exacerbation of the urticarial rash ensued but was quickly resolved upon administration of methylprednisolone the following day.

On the fourth day of hospitalization, as the patient's symptoms had completely resolved, a steroid taper was initiated. The methylprednisolone dose was adjusted to 40 mg IV once daily, with continued administration of cetirizine and diphenhydramine. By the fifth day, following the complete resolution of symptoms and histopathological confirmation of NUD, the patient was deemed suitable for discharge. The prescribed outpatient regimen included cetirizine 10 mg twice daily, colchicine 0.6 mg twice daily, famotidine 20 mg twice daily, and a prednisone taper starting at 40 mg daily (administered as four 10 mg tablets daily for seven days), with subsequent weekly dose reductions (Table 1).

**Table 1:** Clinical timeline of presentation, diagnostic workup, and treatment response. This table outlines the chronological sequence of the patient's symptoms, clinical findings, interventions, and responses from initial onset through hospital discharge. It includes details of rash progression, systemic manifestations, diagnostic evaluations, and therapeutic adjustments. The timeline emphasizes the refractory nature of the disease to standard treatments and the subsequent clinical improvement following targeted interventions.

Timeframe	Event/Clinical Findings	Treatment	Response
Month 0	Initial onset: intermittent pruritus localized to the chin and left shoulder; no clear trigger	Montelukast 4 mg daily × 1 week	Complete symptom resolution for 1 month
Months 1–7	Five recurrent episodes of pruritus with progressive erythematous papules → rash spreading from chin to shoulder, trunk, thighs, and upper extremities; hyperpigmentation after episodes; photosensitivity; burning sensation	Montelukast retrial	No relief
Month 8 (Early)	Rash progressed to diffuse wheals (upper extremities, neck, buccal area, trunk)	2.5% topical hydrocortisone cream × 2 weeks	No improvement
Month 8 (Late)	Rash extended to bilateral hands; new angioedema of the upper lip; joint pain in wrists, MCP, and PIP joints	None (ED visit)	Progression
Hospital Day 0	Admission: afebrile, diffuse urticarial rash with angioedema; labs: CRP 4.3, weakly +ANA; biopsy: dense dermal neutrophilic infiltrate without vasculitis → NUD diagnosis	IV methylprednisolone 20 mg daily; diphenhydramine 25 mg IV q8h	Minimal improvement
Hospital Day 2	Persistent symptoms	Increased methylprednisolone to 20 mg IV TID; added cetirizine 10 mg BID	Improvement begins
Hospital Day 3	Methylprednisolone held to trend leukocytes → transient rash flare	Restarted methylprednisolone	Rash resolved rapidly
Hospital Day 4	Symptom-free	Began steroid taper: methylprednisolone 40 mg IV daily + continued cetirizine and diphenhydramine	Maintained resolution
Hospital Day 5	Discharge	Cetirizine 10 mg BID, colchicine 0.6 mg BID, famotidine 20 mg BID, prednisone taper starting at 40 mg daily	Stable at discharge

## Discussion

NDs are a broad category of inflammatory skin disorders characterized by the accumulation of neutrophils in various layers of the skin and subcutaneous tissue. NUD is a rare subset of dermal NDs, distinct from chronic urticaria due to its histopathological findings and its association with systemic autoinflammatory diseases [5]. Establishing an accurate diagnosis of NUD can be challenging, given its overlap with conditions such as urticarial vasculitis, SLE, and Still’s disease.

Clinically, NUD presents as transient, erythematous macules, papules, or plaques that resemble urticaria but lack responsiveness to antihistamines [6]. Unlike conventional urticaria, NUD is often accompanied by systemic symptoms such as fever, arthralgia, and fatigue [7]. In our case, the patient exhibited a progressive eruption of urticarial lesions with episodes of angioedema, an uncommon but reported feature of NUD. Additionally, her symptoms were refractory to initial treatment with antihistamines and corticosteroids, necessitating further diagnostic investigation.

Histopathological analysis plays a crucial role in differentiating NUD from other urticarial syndromes. The characteristic findings include a perivascular neutrophilic infiltrate without leukocytoclastic vasculitis, features that were observed in our patient’s biopsy [2, 8]. While direct immunofluorescence testing was negative, further reinforcing the absence of urticarial vasculitis, the presence of systemic inflammatory markers such as an elevated C-reactive protein and weakly positive ANA raised suspicion of an underlying autoinflammatory disorder. These findings support prior literature indicating that NUD often occurs in the context of systemic diseases such as SS, CAPS, or AOSD [8, 9].

Treatment of NUD remains challenging due to its resistance to conventional urticaria therapies. Systemic corticosteroids can provide temporary symptom relief in patients with NDs but do not achieve sustained remission and carry significant long-term adverse effects [3]. Neutrophil-targeting agents such as colchicine and dapsone have been reported as effective alternatives in previous studies [7, 10, 11]. In our case, colchicine was initiated following hospital discharge, leading to a brief period of one month with symptom resolution. However, the patient reported symptomatic recurrence following steroid cessation, including facial swelling, erythema, and urticaria. In this case, disease recurrence upon steroid tapering indicated that an underlying systemic process was contributing to disease persistence.

Given the patient’s refractory symptoms, the treatment approach was escalated to immunomodulatory therapy with cyclosporine and omalizumab, which resulted in partial but meaningful symptomatic improvement and a reduction in pruritic symptoms with persistence of erythema and edema. This aligns with emerging evidence supporting the use of IL-1 and TNF-alpha inhibitors in cases of NUD associated with autoinflammatory conditions [12]. Genetic testing for mutations associated with CAPS and related syndromes may provide further insights into the pathogenesis of NUD and assist in selecting more targeted therapies in refractory cases.

## Conclusions

NUD is a rare and underrecognized condition that can mimic chronic urticaria, leading to delays in diagnosis. This case underscores the importance of histopathological evaluation and a multidisciplinary approach in managing refractory cases, especially when associated with underlying autoinflammatory disorders. The partial response to immunosuppressive and biologic agents highlights the potential need for targeted therapies in select patients.
